# Author Correction: Changes in gene expression predictably shift and switch genetic interactions

**DOI:** 10.1038/s41467-019-12350-y

**Published:** 2019-09-17

**Authors:** Xianghua Li, Jasna Lalić, Pablo Baeza-Centurion, Riddhiman Dhar, Ben Lehner

**Affiliations:** 1 Centre for Genomic Regulation (CRG), The Barcelona Institute of Science and Technology, Dr. Aiguader 88, Barcelona, 08003 Spain; 2 Universitat Pompeu Fabra (UPF), Barcelona, Spain; 3 ICREA, Pg. Luis Companys 23, Barcelona, 08010 Spain

**Keywords:** Genetics, Systems biology, Computational models, Epistasis

Correction to: *Nature Communications* 10.1038/s41467-019-11735-3, published online 29 August 2019.

The original version of this Article contained an error in the spelling of the author Jasna Lalić, which was incorrectly given as Jasna Lalic. This has now been corrected in both the PDF and HTML versions of the Article.

