# Supplementary material for: Changes in gene expression predictably shift and switch genetic interactions
Source: Nat Commun. 2019 Aug 29;10:3886. doi: 10.1038/s41467-019-11735-3 (PMC6715729; doi:10.1038/s41467-019-11735-3)
Supplement: Supplementary file 1 — Supplementary Information [file 41467_2019_11735_MOESM1_ESM.pdf]

Supplementary Information

Changes in gene expression predictably shift and switch genetic interactions

Li et al.

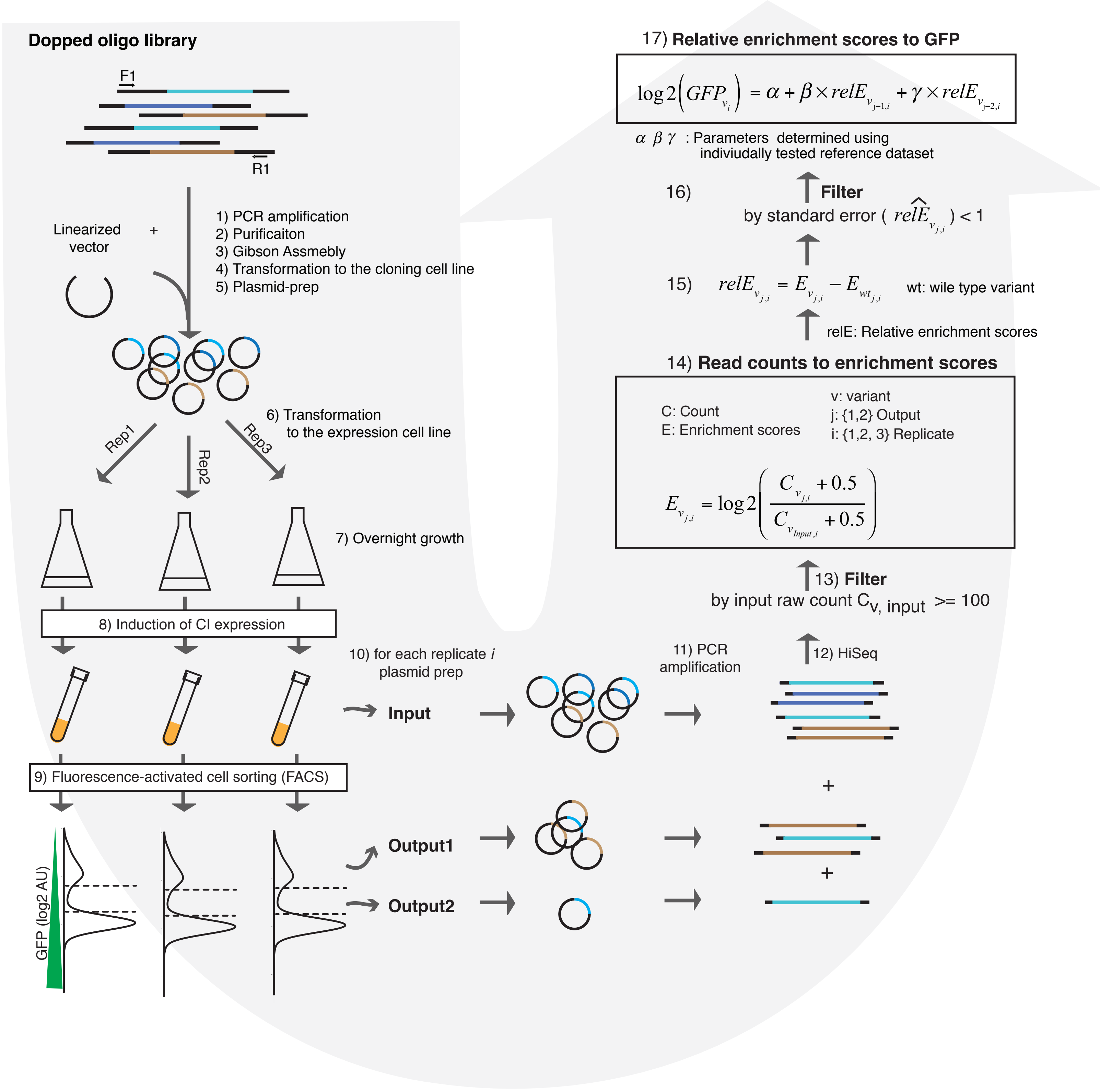

Supplementary Figure 1. Experimental pipeline.

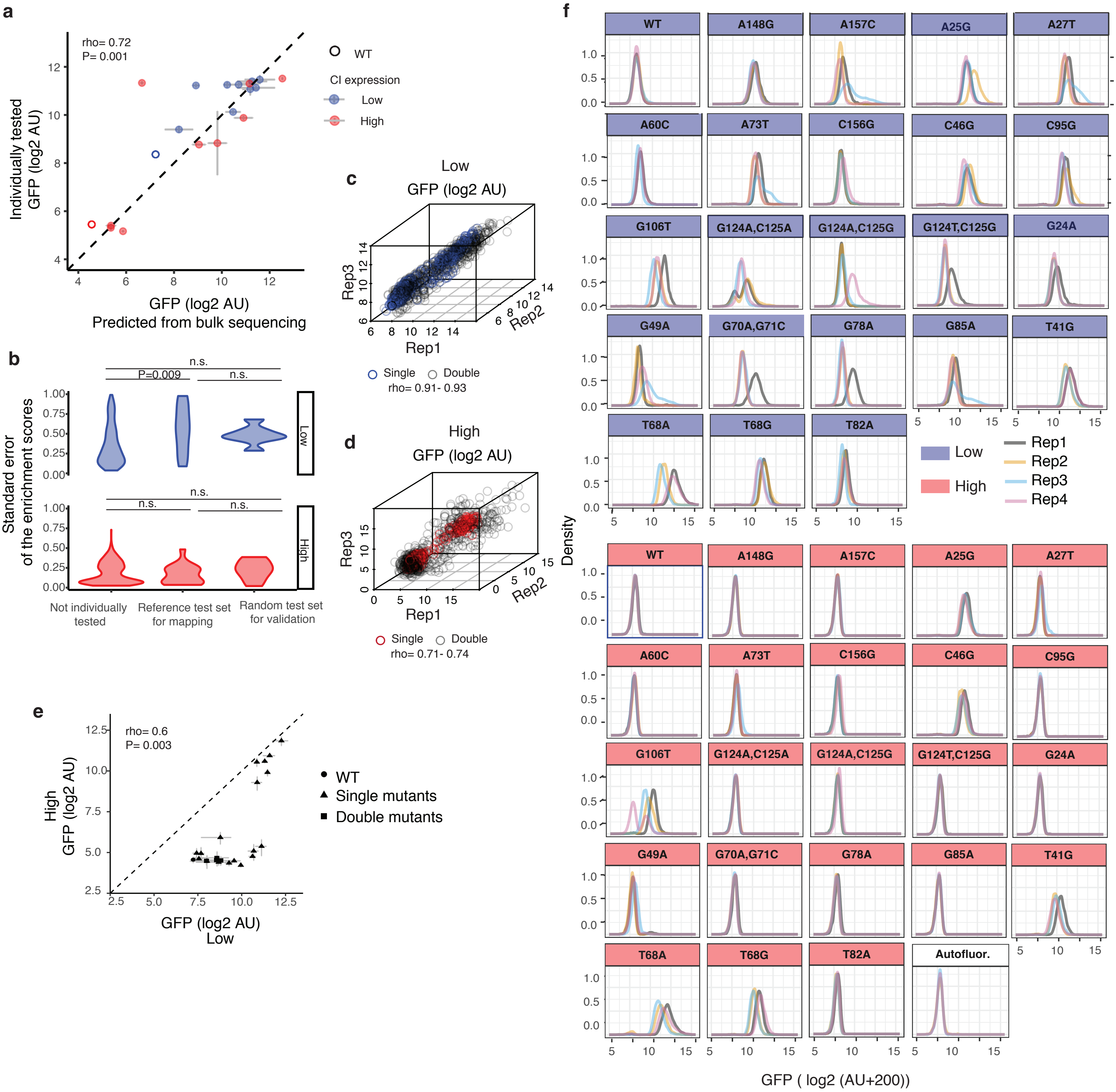

Supplementary Figure 2. Reproducibility of mutational effects between biological replicates.
(a) Correlation of target gene expression estimated by deep sequencing, with target gene expression individually quantified for a second validation dataset of 9 single and double mutants at low and high expression levels together with wild type. Error bars denote standard error of the mean from three biological replicates.
(b) Violin plots of the standard error for enrichment scores from sequencing data for the analysed datasets (n=1182), reference dataset as shown in Figure 1d (n=22, excluding wild type variant) and the independent validation dataset as shown in the panel (a) (n=9). Groups compared using Kruskal-Wallis test with post hoc Dunn's test.
(c and d) Spearman correlations of mutational effects among three biological replicates for low (c) and high (d) CI expression.
(e) Comparisons of mutational effects between low and high expression level for 22 individually retested single and double mutants together with wild type. Error bars denote standard error of the mean.
(f) Density plots of GFP expression for the 22 individually re-tested single and double mutants at the two expression levels of CI.

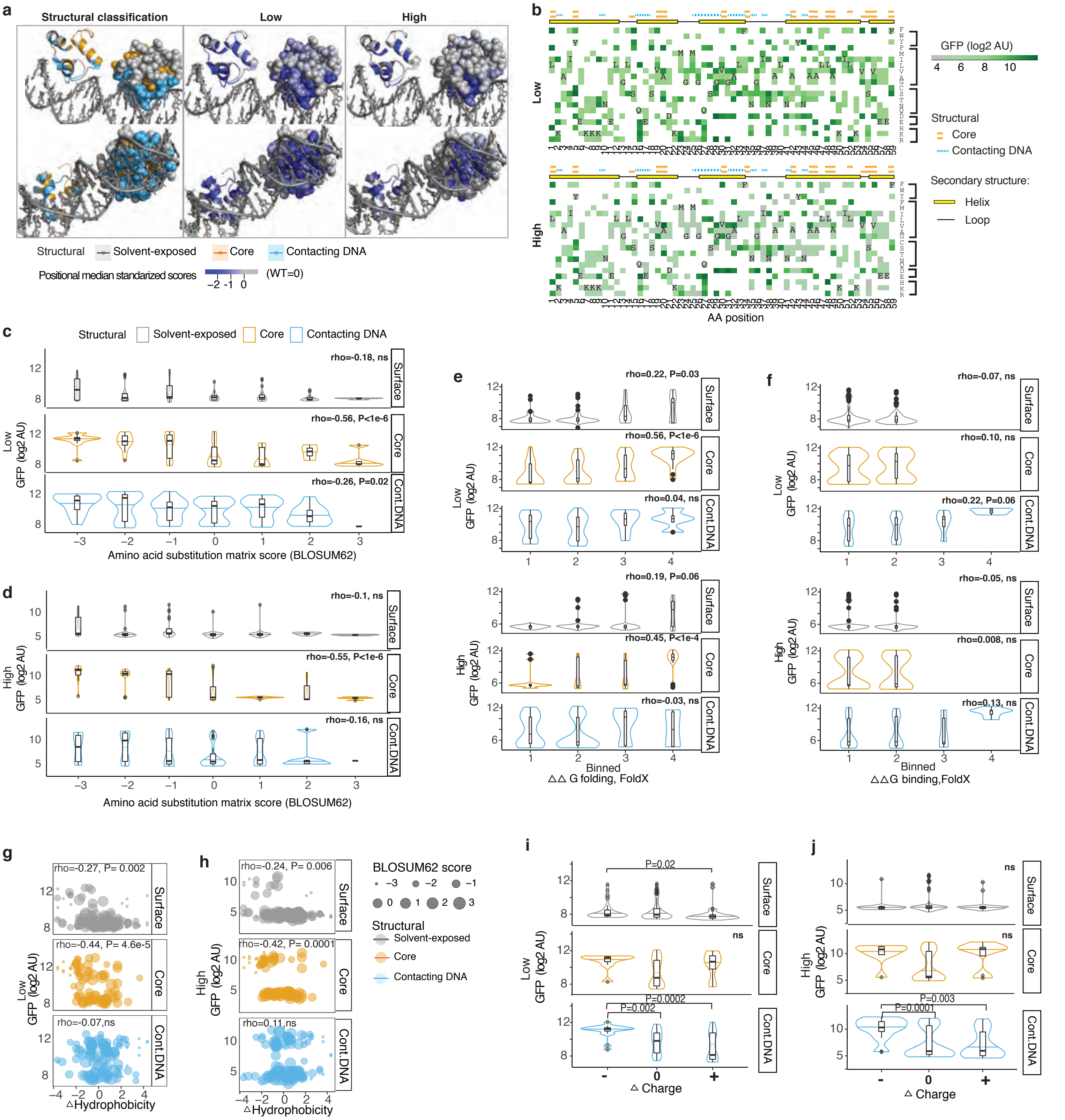

Supplementary Figure 3. Mutational effects depend on both the chemical features of amino acid substitutions and the tertiary structural positions.
(a) Structure of CI dimer bound to an operator (PDB 3bdn). One monomer is shown as a ribbon and the other one with all its atoms shown as spheres. Only the mutagenized HTH domain is shown. Left panel is the structural classification of the residues. Middle and right panels show the positional median z-scores of GFP expression levels after subtracting wild type z-scores at the two expression levels of CI. Z-scores rather than absolute GFP expression levels are shown here to compare positional sensitivity to mutations at two expressions of CI. (b) Heatmaps of mean GFP expression for single mutations at the two expression levels. Amino acids are ordered based on their similarities, from top to bottom: hydrophobic aromatic (F,W,Y), hydrophobic nonpolar aliphatic (P,M,I,L,V,A,G), hydrophilic polar uncharged (C,S,T,N,Q), hydrophilic negatively charged (D,E) and hydrophilic positively charged (H,K,R). Wild type amino acids are shown as letters inside the heatmap. (c and d) Target GFP expression compared to the amino acid substitution matrix scores (BLOSUM62) at low (c) and high (d) expression of CI. (e and f) Target GFP expression compared to the binned FoldX-predicted changes in the folding energy of the protein (e) and protein-DNA binding (f) at the two expression levels. Bin1 corresponds to ΔΔG≤0; Bin2: 0<ΔΔG≤2.5; Bin3 : 2.5<ΔΔG≤5; and Bin4: ΔΔG>5. (g and h) Target gene expression compared to the change in the hydrophobicity at low (g) and high (h) expression of CI. (i and j) Target gene expression compared to changes in the side chain charges at low (i) and high (j) expression of CI. Classes compared using Kruskal-Wallis test with post hoc Dunn's test. All P-values were Bonferroni adjusted.

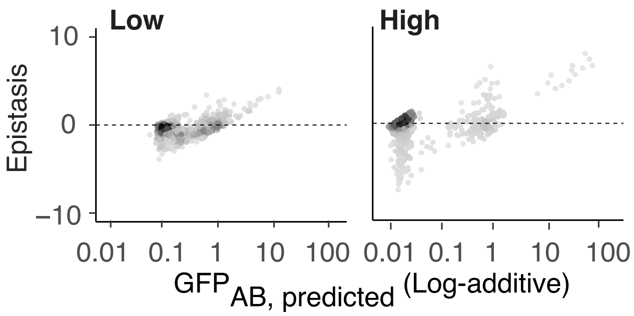

Supplementary Figure 4. Epistasis versus GFP expression levels for observed data.

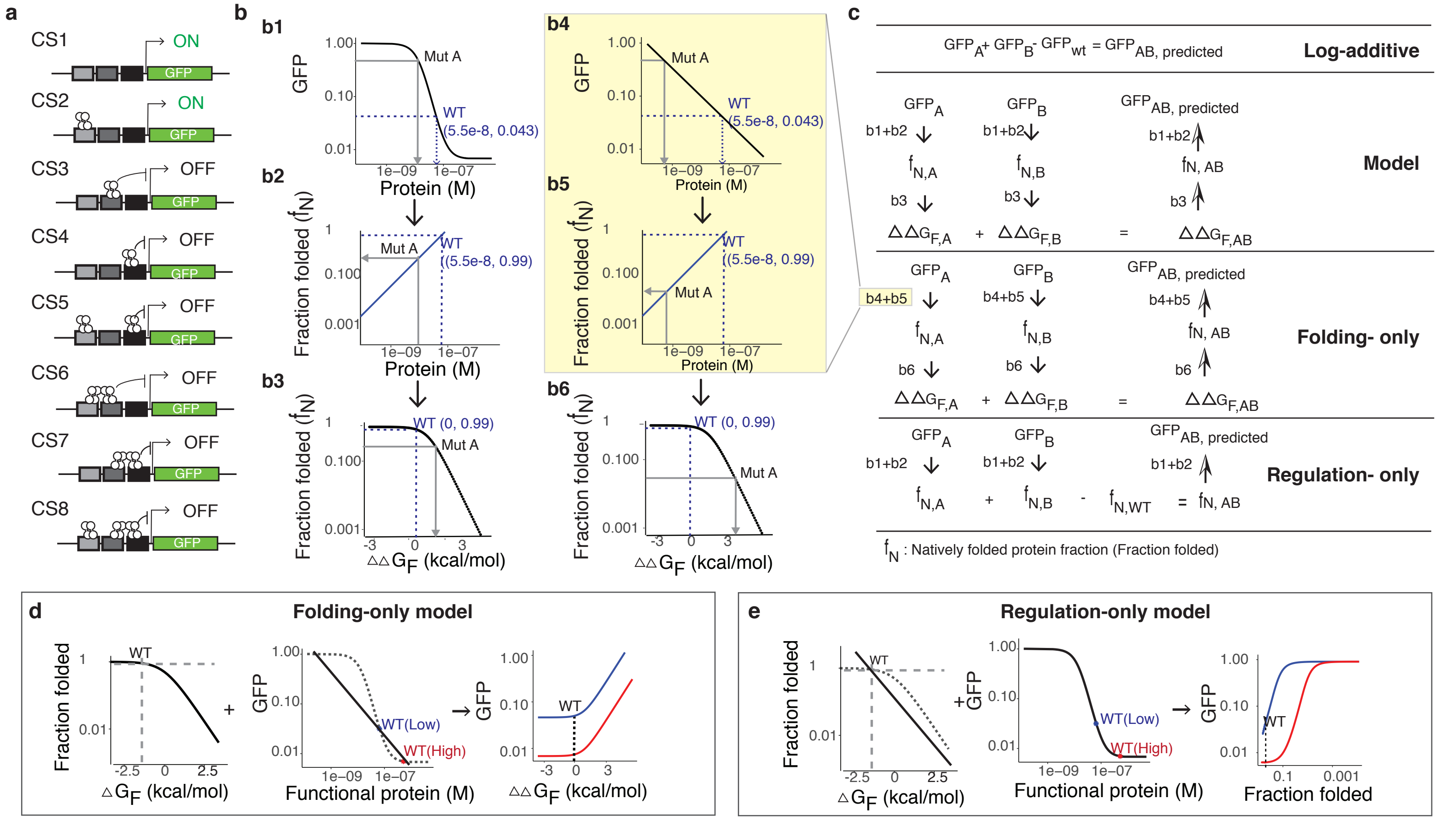

Supplementary Figure 5. Mathematical models.
(a) Eight configuration states (CS) of the PR promoter. (b) Obtaining functional protein concentration (panels b1,b4), fraction of folded protein (panels b2,b3), and change in folding energy (panels b2,b3) from GFP expression levels of a mutation at low expression of the protein. (c) Scheme for predicting double mutants' GFP expression levels from single mutants' GFP expression levels based on different models. (d) Folding-only model. (e) Regulation-only model.

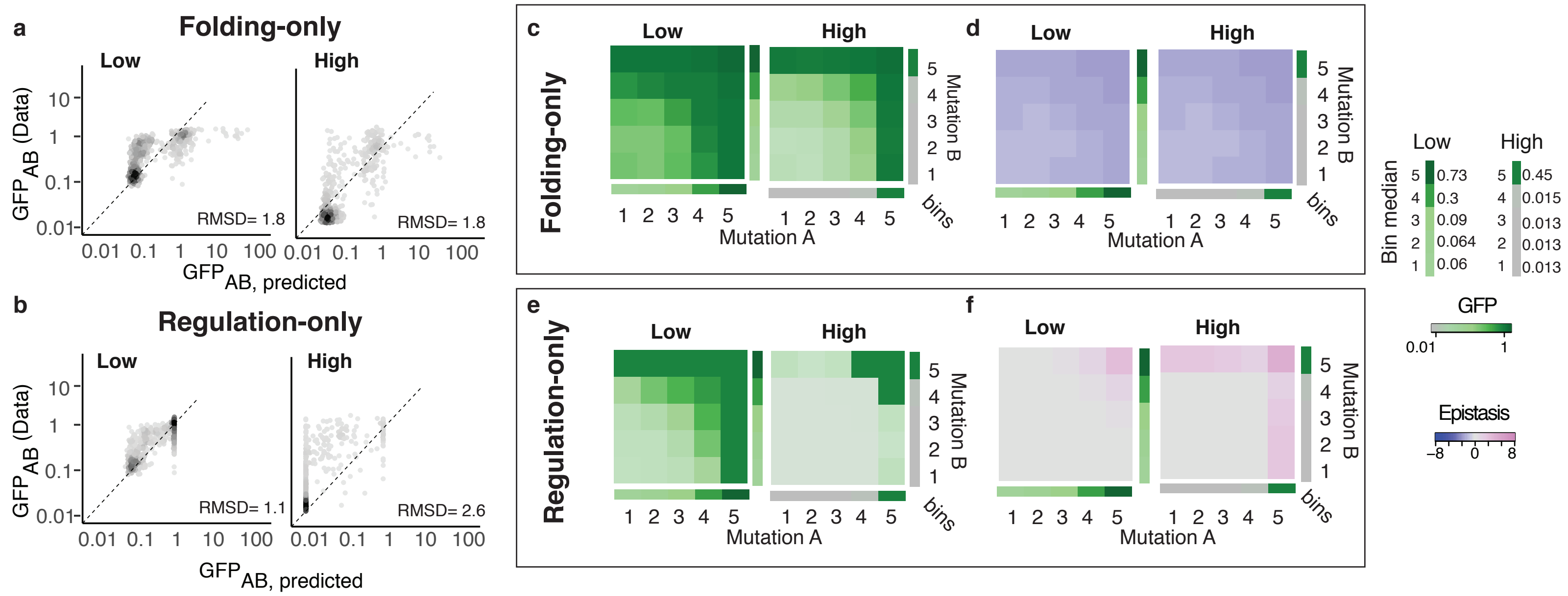

Supplementary Figure 6. Predictions of double mutants based on folding-only or regulation-only model.
(a and b) Observed versus predicted GFP expression levels for the folding-only (a) and regulation-only (b) models. RMSD: root-mean-square-deviation from the predicted to the observed data. (c – f) Binned median target gene expression levels (c, e) and epistasis scores (d, f) for the folding-only (c, d) and regulation-only (e, f) models. Mutations were sorted into 5 equally populated bins by their single mutant phenotypes as in Figure 3j,k.

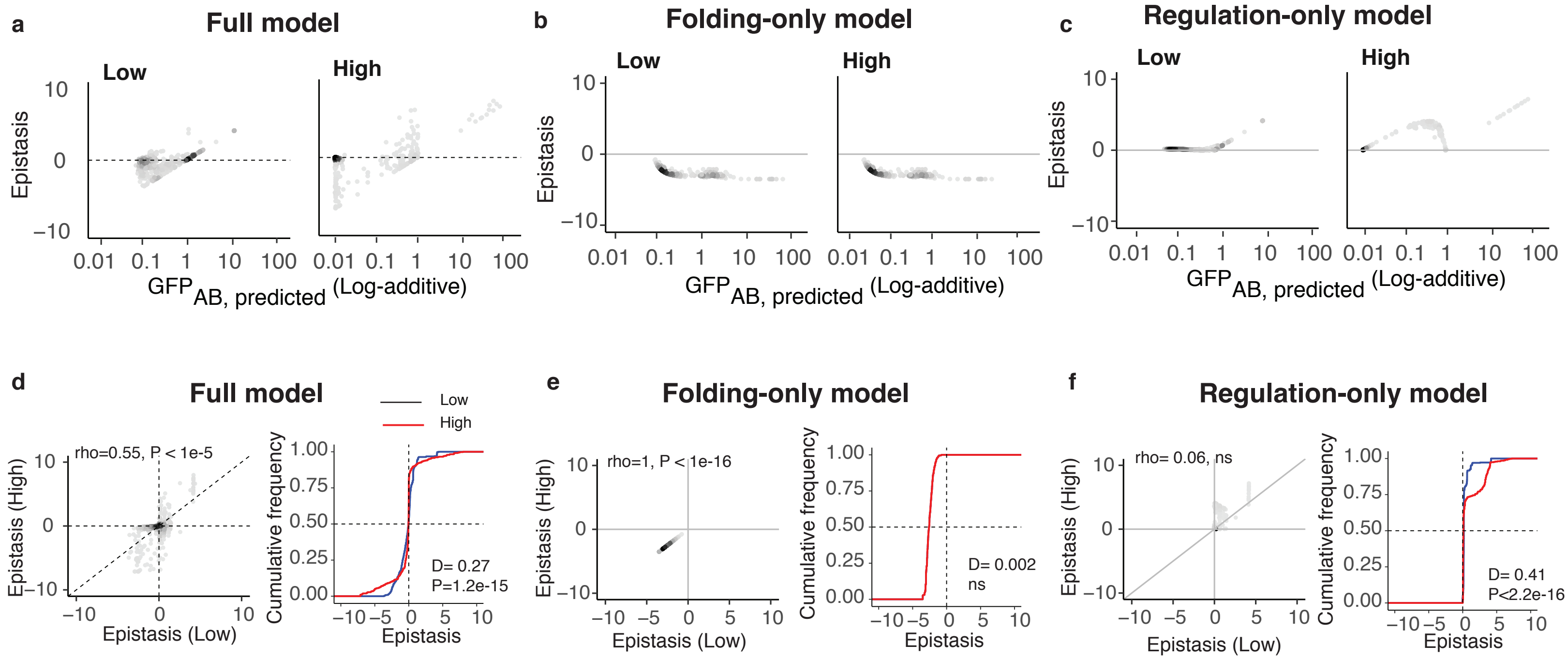

Supplementary Figure 7. Epistasis pattern predicted from different models.
(a – c) Epistasis versus GFP expression levels predicted from full model (a), folding-only model (b) and regulation-only model (c). (d – f) Epistasis scores at the two expression levels of CI protein for full model (d), folding-only model (e) and regulation-only model (f). Two-sample Kolmogorov–Smirnov test was performed for cumulative distributions of epistasis scores at the two expression levels of CI protein.

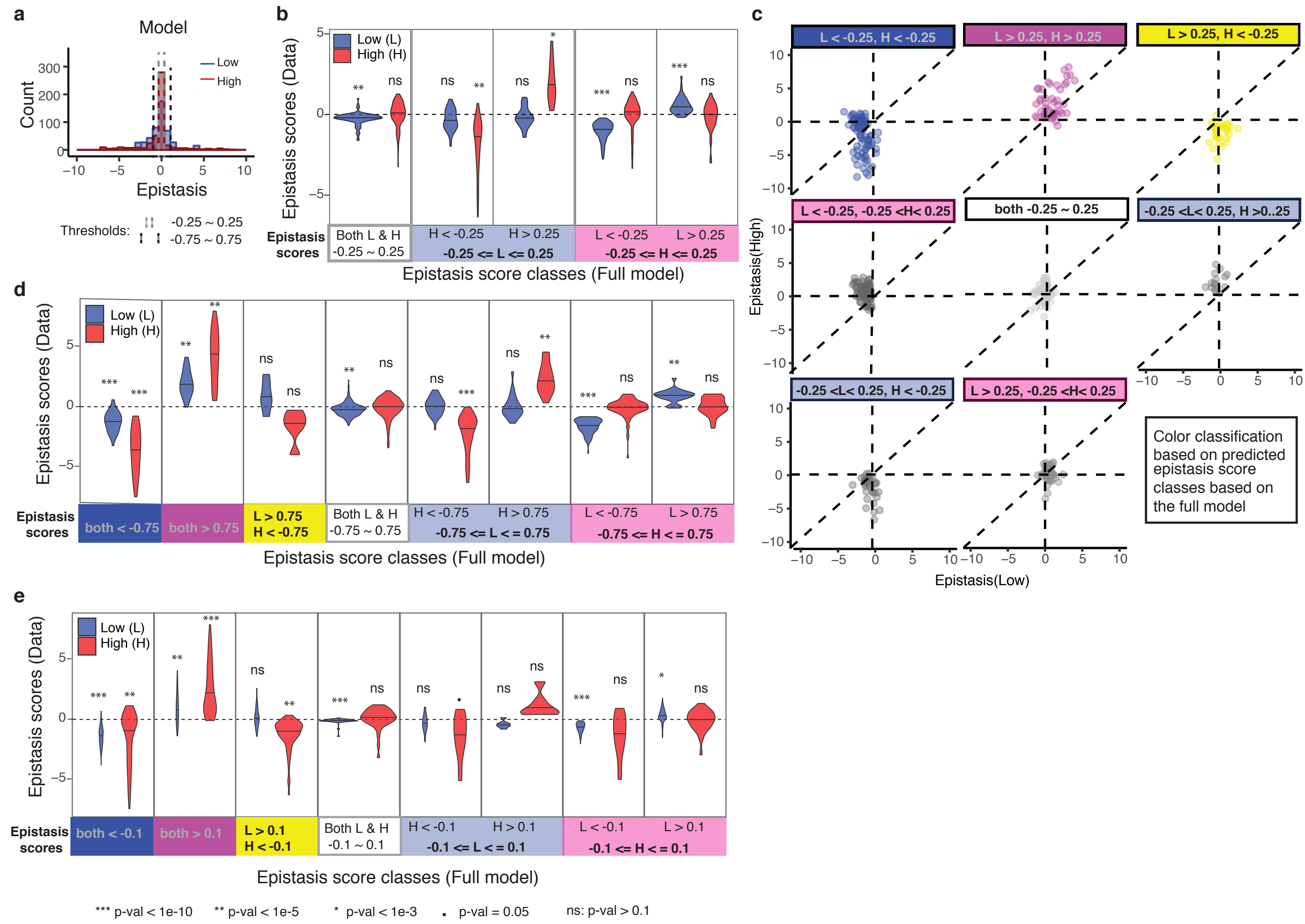

Supplementary Figure 8. Observed versus predicted expression level-dependent changes in epistasis.
(a) Histogram of the model-predicted epistasis score distributions at the two expression levels of the protein. The grey dotted lines mark the centre bin with the epistasis score thresholds of -0.25 and 0.25; and the black dotted lines mark the centre three bins with the epistasis score thresholds of -0.75 and 0.75. (b and c) Distribution of the observed epistasis scores grouped by the model-predicted classes of epistasis scores, with classification threshold of -0.25 and 0.25. (d and e) Distribution of the observed epistasis scores grouped by the model-predicted classes of epistasis scores, with two additional classification thresholds, between -0.75 and 0.75 (d) and between -0.1 and 0.1 (e). “L” - low expression and “H” - high expression.The one-sample Wilcoxon signed rank test was performed to test whether average epistasis scores are significantly different from 0. P-values are adjusted with Bonferroni multiple test correction method.

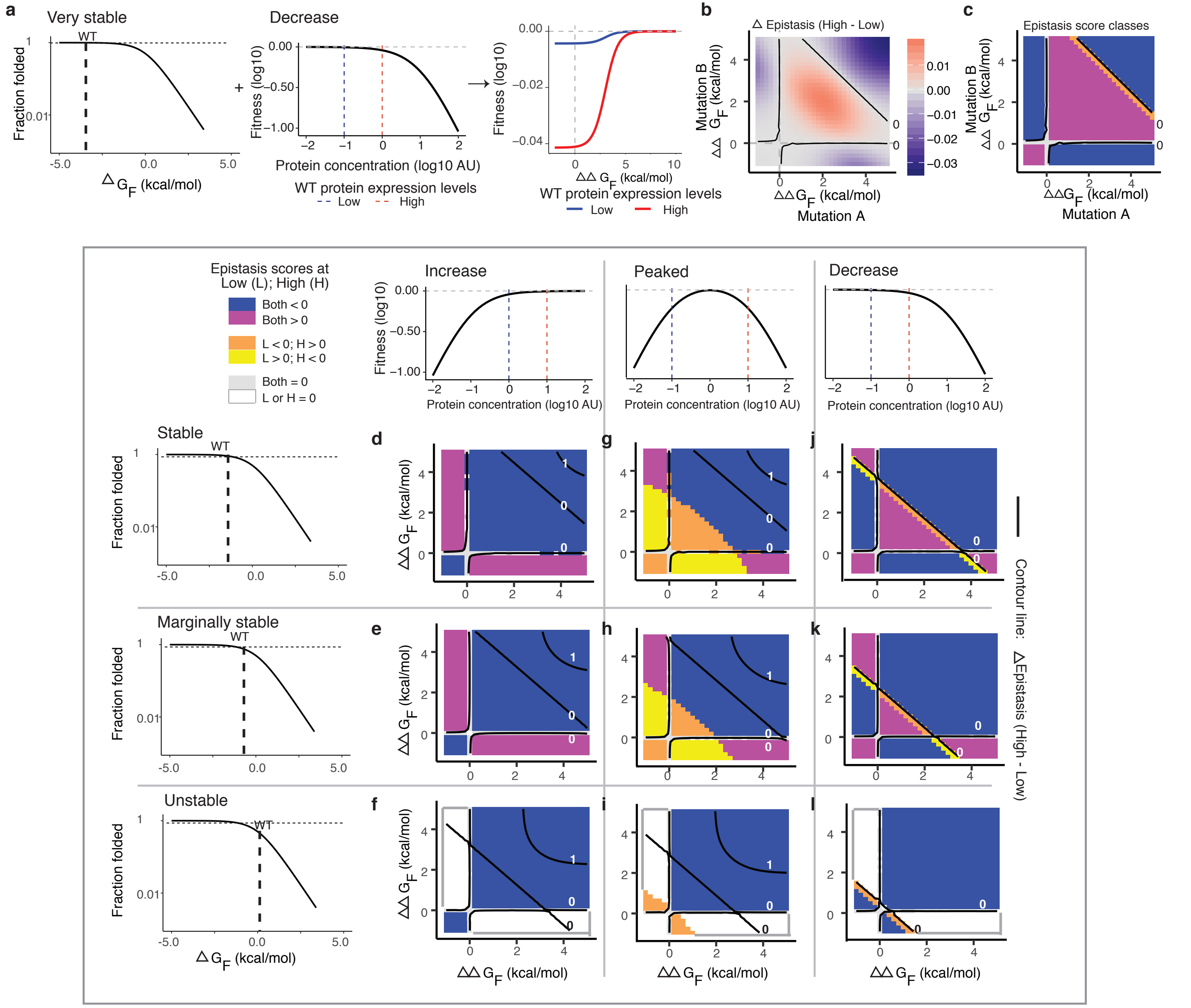

Supplementary Figure 9. Concentration-dependent genetic interactions in the yeast fitness landscape. (a – c) Concentration-dependent mutation effects and epistasis in a “decreasing” expression-fitness function54. (d – l) Concentration-dependent epistasis for three common expression-fitness functions with stable, marginally stable and unstable proteins.

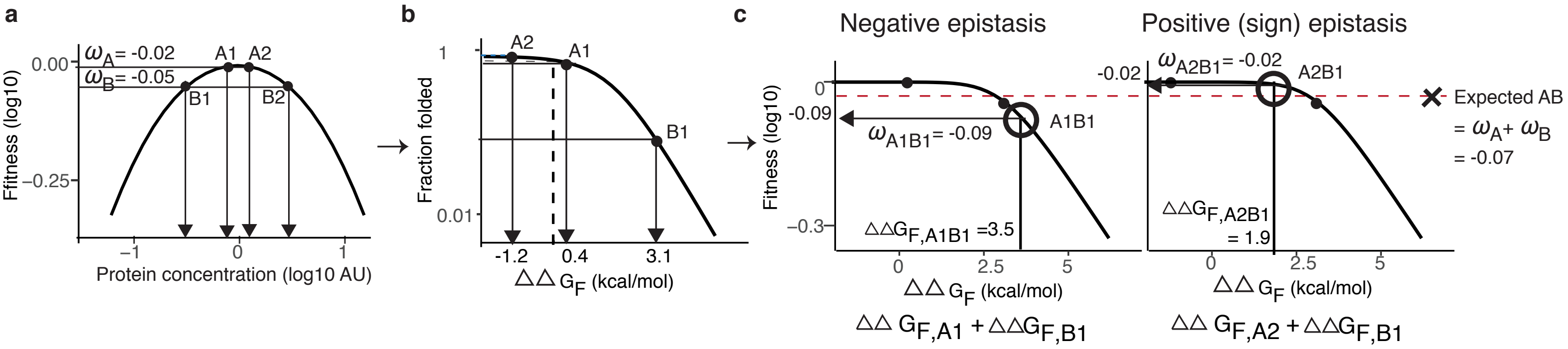

Supplementary Figure 10. Unpredictable double mutant phenotypes.
(a) A measured fitness effect can be caused by two different changes in protein concentration in a 'peaked' fitness landscape when the WT protein is expressed at the fitness optimum. (b) Only very small changes in fitness can be mapped to either increased or decreased fraction of folded protein, due to the limit of fraction of folded protein (maximum equals to 1). For example, a mutant with the fitness effect of -0.02 (ωA) can be caused by two different mutations (A1 and A2) that cause changes in the free energy of protein folding (ΔΔGF,A1 or ΔΔGF,A2) and so two different changes in protein concentration. In contrast, larger fitness changes can only be caused by one change in free energy of folding. For example, a mutant with a fitness effect of -0.05 (ωB) can be caused by either a 5-fold increase or decrease in the functional protein concentration. However, a 5-fold increase in concentration cannot be achieved by a change in folding because it would require more than 100% of the protein to be folded. Therefore, a mutant with a fitness effect of -0.05 can only be caused by a decrease in protein stability (mutant B1). (c) Combining two mutations of known fitness can lead to two possible double mutant outcomes and either positive or negative epistasis. For the case of A2 + B1, mutant A2 is detrimental in the wild type background (ωA2=-0.02), but beneficial at the mutant B1 background (ωA2B1- ωB1= -0.02 –(-0.05)= 0.03). The interaction between mutant A2 and B1 is. Therefore an example of sign epistasis. The possible outcomes are up to 4 if the fitness landscape is not symmetrical.

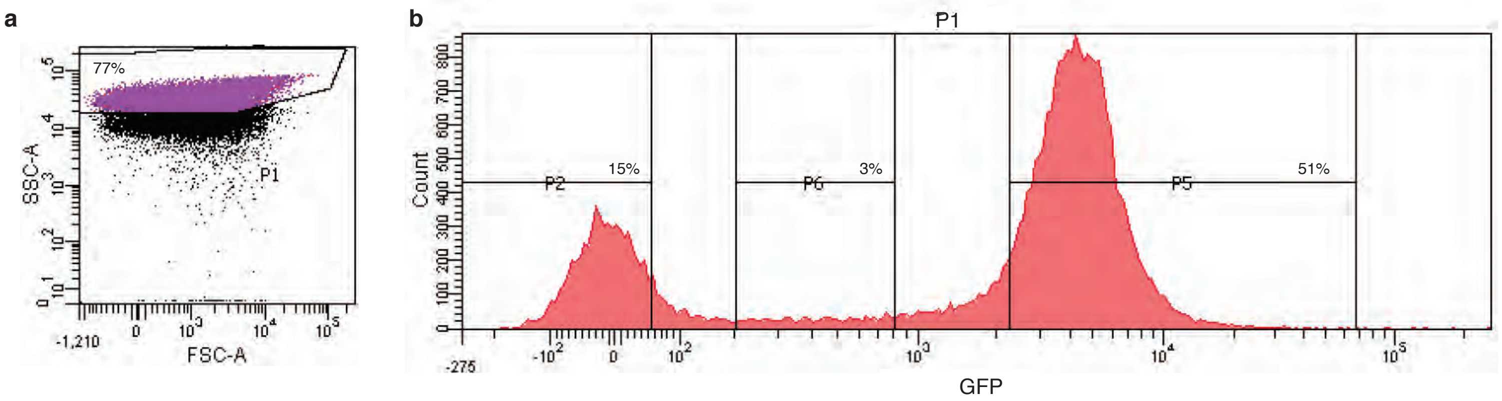

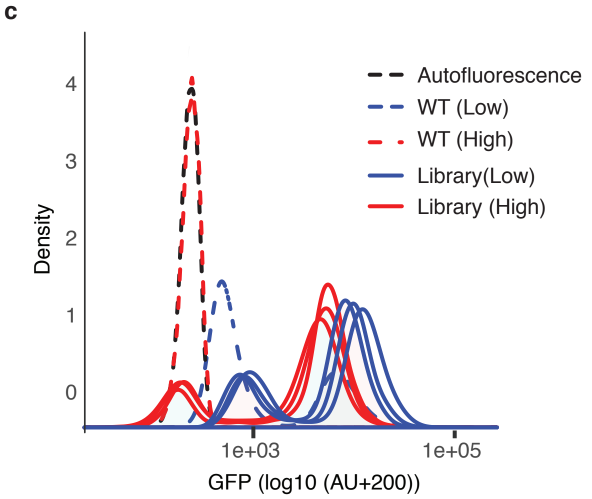

$$\text{GFP\_index} = \frac{\text{Mean (No\_repression)- Mean (wt\_repression)}}{\text{2 X SD (wt\_repression)}}$$

GFP_index

	replicate 1	replicate 2	replicate 3
Library (Low)	0.69	0.68	0.75
Library (High)	1763	1974	924

Supplementary Figure 11. Fluorescence-activated cell sorting (FACS).
(a and b) An example (High expression, replicate 3) of the gating strategy for FACS. Gate P2 and P6 correspond to Output 1 and Ouptut2 in Figure 1c respectively. (c) FACS recordings from each biological replicate performed on different days. GFP_index is used to quantify variation in fluorescence readings between batches.

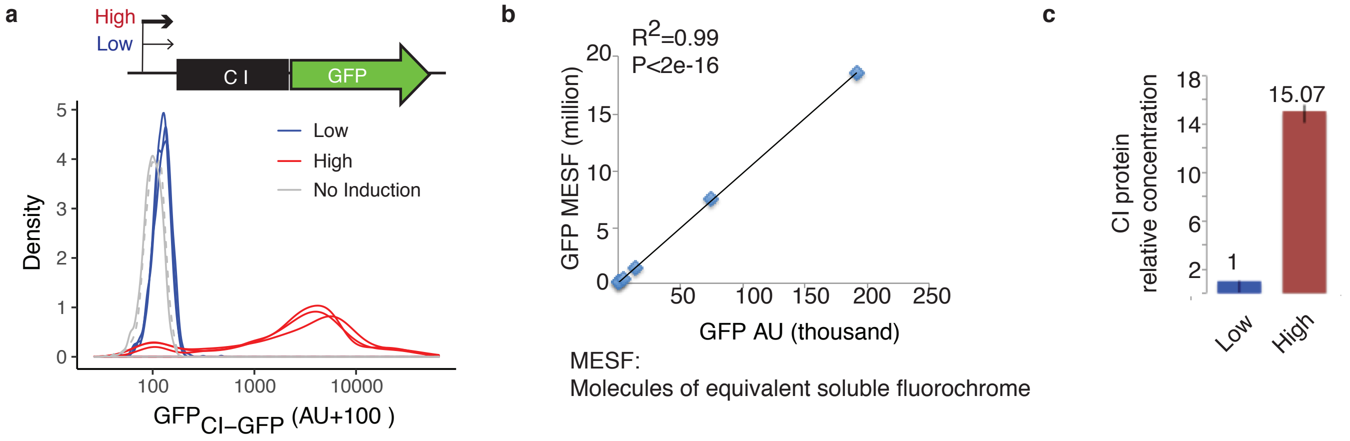

Supplementary Figure 12. Protein quantification.
(a) Distribution of fluorescence signal of cells expressing C-terminal GFP-tagged CI at high and low expression levels. (b) Fluorescence linearly correlates with the number of molecules of equivalent soluble fluorochrome (MESF) from GFP beads. (c) Relative fold-change of soluble CI protein concentrations at high versus low expression levels. Error bars denote standard error of the mean.

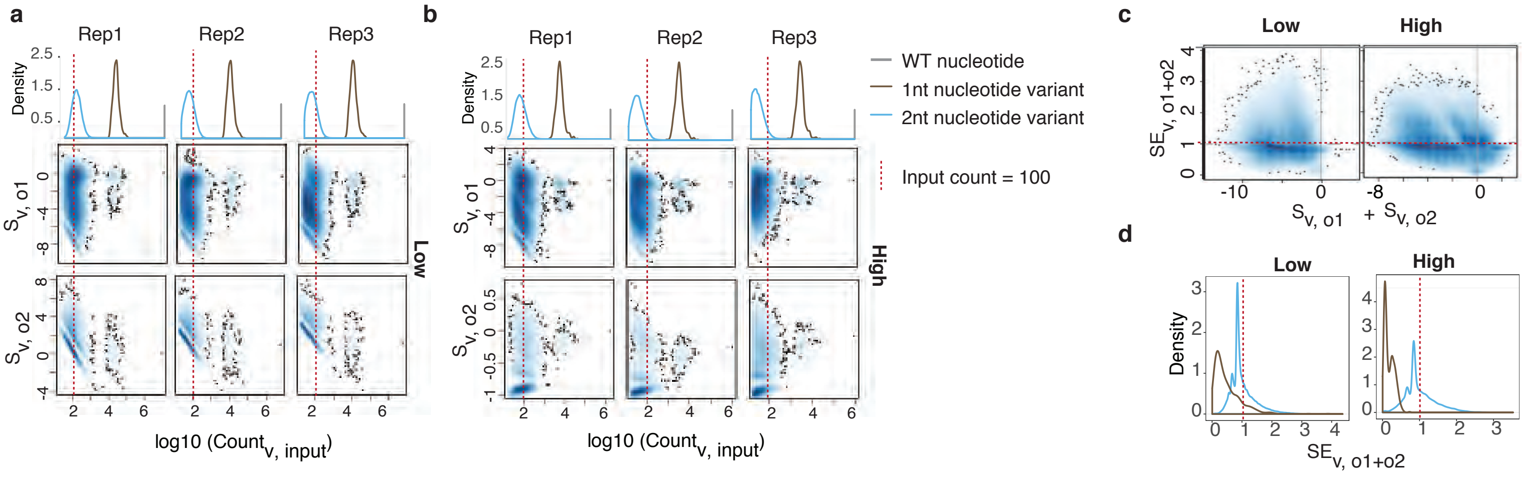

Supplementary Figure 13. Filtering of sequencing data.
(a and b) Sequencing data was filtered to only retain genotypes with at least 100 read counts (red line) in all three biological replicates for both low (a) and high (b) expression datasets. Each smooth scatter panel shows the relationship between enrichment scores (Sv,o1 for Output1 and Sv,o2 for Output2) and input read counts for each replicate. The top density plot shows the input count distribution for each replicate. (c and d) Only variants with propagated mean enrichment score standard errors smaller than 1 (red line) were retained.

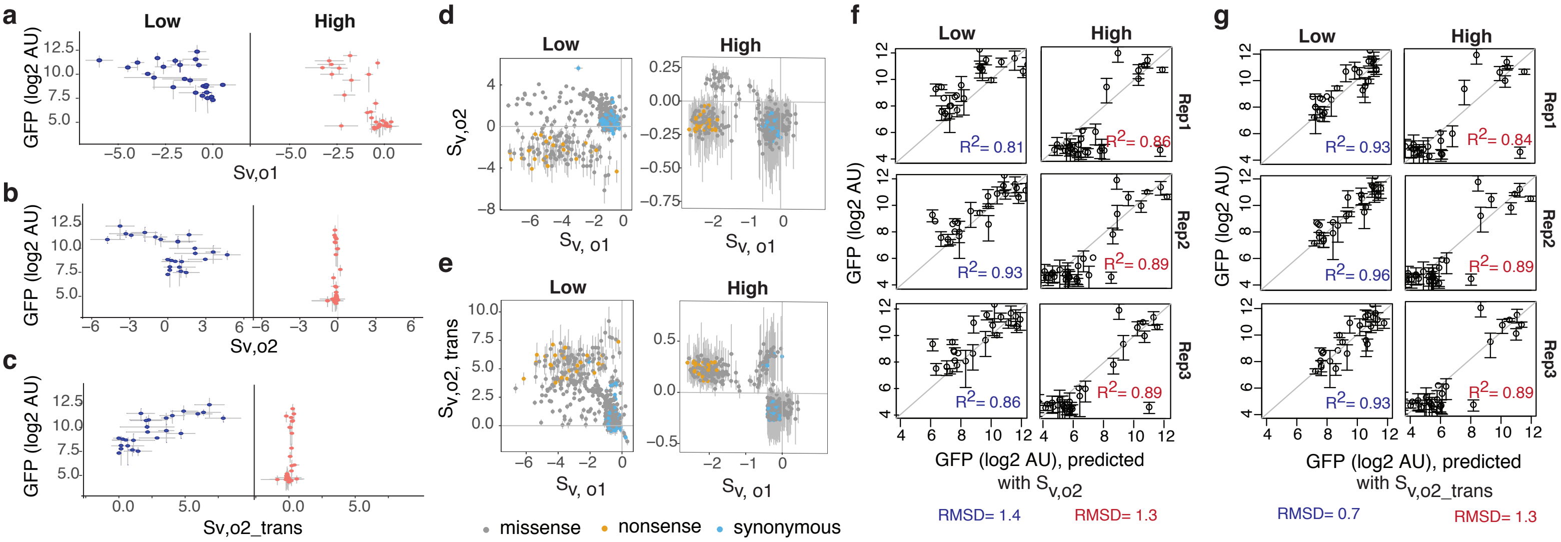

Supplementary Figure 14. Converting enrichment scores to GFP expression.
(a – c) Relationship between GFP signals either with Output1 enrichment scores (a), with Output2 enrichment scores (b), or with transformed Output2 enrichment scores (c) for the individually tested variants (n=23). (d and e) Relationship between Output1 and Output2 enrichment scores (d) or transformed Output2 enrichment scores (e) for all single nucleotide variants (n=531). (f and g) Comparisons of individually tested mean GFP signals with the predicted mean GFP signals from Output1 and Output2 enrichment scores (f) or with Output1 and transformed Output2 enrichment scores (g) (n=23). All error bars denote standard error of the mean. RMSD: root-mean-square-deviation between the predicted and observed data, after averaging the replicates.

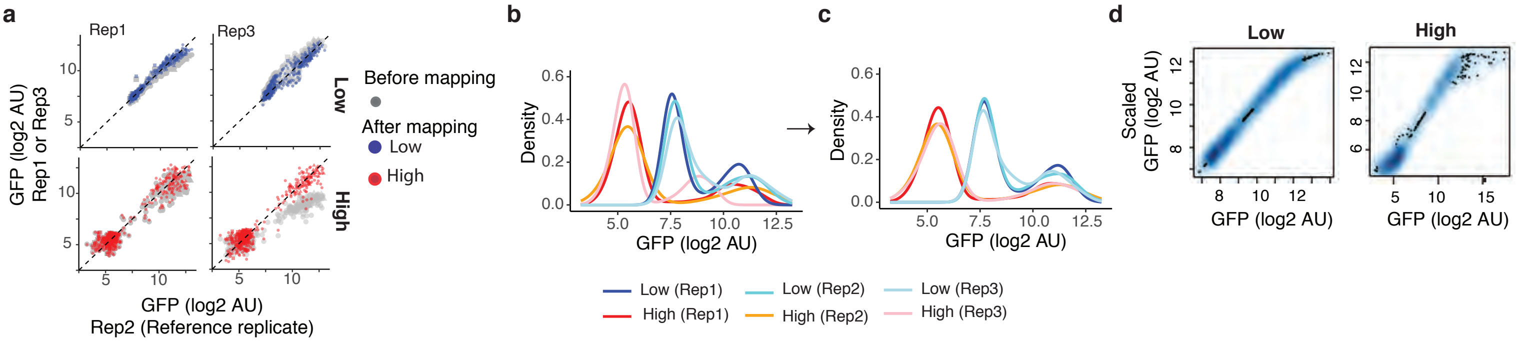

Supplementary Figure 15. Correcting for technical biases.
(a) Relationship between predicted GFP expression for biological replicates for all single nucleotide variants (n=531) before (grey) and after (blue or red) transforming the replicate 1 and 3 data to the reference replicate 2 (see Methods). (b and c) Density plot of GFP expression before (b) and after (c) correcting for technical biases by transforming replicates 1 and 3 to the reference replicate 2 for all single nucleotide variants (n=531). (d) Smooth scatter showing the relationship between the mean GFP signal of all amino acid genotypes (n=888) before and after scaling to the detection range (see Supplementary Methods).

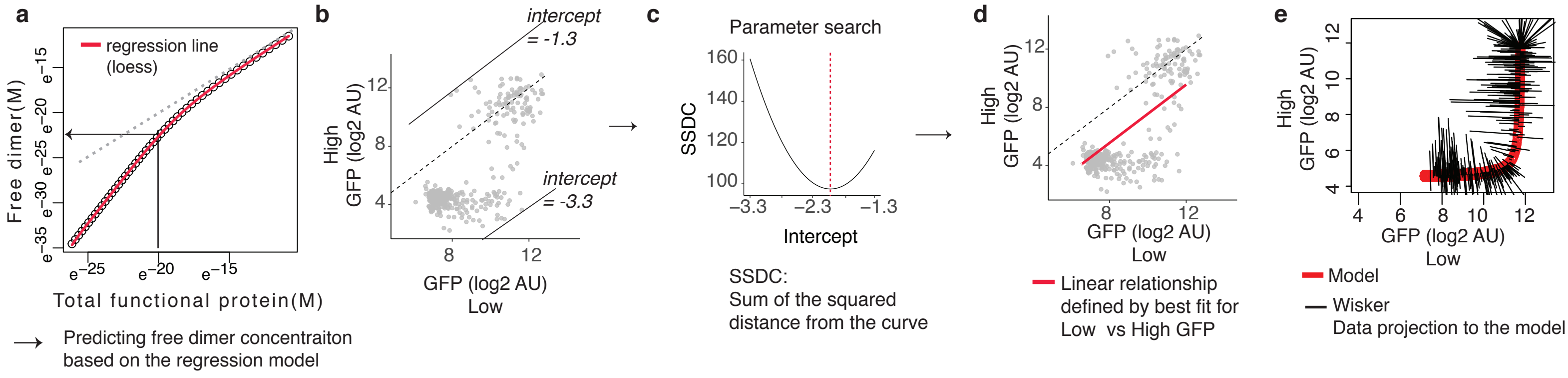

Supplementary Figure 16. Mathematical modelling.

(a) Relationship between free CI dimer concentration and total CI concentration in the cell in Ackers' model. (b – d) Parameter search for the line intercept that best describes the relationship of GFP at low and high expression for the folding-only model. Dashed lines in (b) and (d) mark equal GFP level at the two expression levels. Solid lines in (b) mark the range of the intercepts searched for the best fit. Red dashed line in (c) shows the best fit (the smallest SSDC). (e) Projection of individual data points from observed GFP expression levels at low and high CI expression to the model-predicted curve.

Supplementary Table 1. PCR primers

F: Forward; R: Reverse, 5' to 3'		Note
d-CIF	ACACAAGAGCAGCTTGAGGA	“doped” library amplification
d-CIR	ATTTCTCTGGCGATTGAAGG	
pBAD-cI-F	TCCTCAAGCTGCTCTTGTGT	For Gibson Assemble CI to the backbone
pBAD-CI-R	CCTTCAATCGCCAGAGAAAT	
Q5SDM_CIF	ATGAGCACAAAAAAGAAACC	Changing 5prime UTR for expression of CI
Q5SDM_CIR	GGTTAATTCCTCCTGTTAG	
CI_GFP_fuse_F	GCTGGTTCTGGCGAATTCATGCGTAAAGGCGAAGAAC	Making CI-GFP fusion for protein quantification
CI_GFP_fuse_R	AGCGGAGCCAGCGGATCCGCCAAACGTCTCTTCAGG	
colony-F1	GGCGTCACACTTTGCTATGC	For colony PCR
colony-R1	ACAGTTCTTCGCCTTTACGC	
dCI_F_s	GCTTGAGGACGCACGTC	“doped” library amplification for sequencing library
dCI_R_s	TCTGGCGATTGAAGGGCT	
d_CI_Fs_1	GAACGTTCGCTTGAGGACGCACGTC	Input.rep1.barcoded
d_CI_Rs_1	GAACGTTCTCTGGCGATTGAAGGGCT	
d_CI_Fs_2	GCCGAATTGCTTGAGGACGCACGTC	Input.rep2.barcoded
d_CI_Rs_2	GCCGAATTTCTGGCGATTGAAGGGCT	
d_CI_Fs_3	CGGCAATTGCTTGAGGACGCACGTC	Input.rep3.barcoded
d_CI_Rs_3	CGGCAATTTCTGGCGATTGAAGGGCT	
d_CI_Fs_4	GCGCATATGCTTGAGGACGCACGTC	Output1 &2.rep1.barcoded
d_CI_Rs_4	GCGCATATTCTGGCGATTGAAGGGCT	
d_CI_Fs_5	CAACCATGGCTTGAGGACGCACGTC	Output1 &2.rep2.barcoded
d_CI_Rs_5	CAACCATGTCTGGCGATTGAAGGGCT	
d_CI_Fs_6	CGTACCTTGCTTGAGGACGCACGTC	Output1 &2.rep3.barcoded
d_CI_Rs_6	CGTACCTTTCTGGCGATTGAAGGGCT	
Q5SDM1_F	TTGATGCCATTAAATAAAGCAC	G124T,C125G
Q5SDM1.1_R	TGCATTAAATcaTTATAACGCCGCATTG	
Q5SDM1_F	TTGATGCCATTAAATAAAGCAC	G124A,C125G
Q5SDM1.2_R	TGCATTAAATctTTATAACGCCGCATTG	
Q5SDM1_F	TTGATGCCATTAAATAAAGCAC	G124A,C125A
Q5SDM1.3_R	TGCATTAAATttTTATAACGCCGCATTG	
Q5SDM4_F	AGACAAGATGacGATGGGGCAGTC	G70A,G71C
Q5SDM4_R	GCGACAGATTCCTGGGAT	
Q5SDM5_F	AGGAATCTGTCGCcGACAAGATGGGGA	A60C (synonymous)
Q5SDM5_R	TCCCCATCTTGTCGGCGACAGATTCCT	
Q5SDM6_F	GCAATTTATGAAAAAAAaAAAAATGAACTTGGCTT	G78A (synonymous)
Q5SDM6_R	AAGCCAAGTTCATTTTTTTTTTTTTTCATAAATTGC	
Q5SDM7_F	CTTGCAAAAATTCTgAAAGTTAGCGTTGAAGAATTTAGC	C156G(synonymous)
Q5SDM7_R	GCTAAATTCTTCAACGCTAACTTTCAGAATTTTTGCAAG	
Q5SDM8_F	AAATGAACTTGGCTgATCCCAGGAATCTGTCG	T41G (nonsense)
Q5SDM8_R	CGACAGATTCCTGGGATCAGCCAAGTTCATTT	
Q5SDM9_F	CTTATCCCAGaAATCTGTCGC	G49A
Q5SDM9_R	CCAAGTTCATTTTTCTTTTTTTC	
Q5SDM10_F	GCAATTTATGAAAAAAAaAAAAATGAACTTGGCTT	G24A
Q5SDM10_R	AAGCCAAGTTCATTTTTTTTTTTTTTCATAAATTGC	
Q5SDM11_F	TCGCAGACAAGAaGGGGATGGGGCAG	T68A
Q5SDM11_R	CTGCCCCATCCCCtTCTTGTCTGCGA	
Q5SDM12_F	AAAAAAAGAAtAATGAACTTGGCTTATC	A27T
Q5SDM12_R	CATAAATTGCTTTAAGGCG	
Q5SDM13_F	TGAAAAAAAGGAAAATGAACTTGG	A25G
Q5SDM13_R	TAAATTGCTTTAAGGCGAC	
Q5SDM14_F	AAAAATTCTCCAAGTTAGCGTTGAAGAATTTAGC	A157C
Q5SDM14_R	GCAAGCAATGCGGCGTTA	
Q5SDM15_F	GGCGTTGGTGgTTTATTTAATGGC	C95G
Q5SDM15_R	TGACTGCCCCATCCCCAT	
Q5SDM16_F	GGGGCAGTCAaGCGTTGGTGC	G85A
Q5SDM16_R	ATCCCCATCTTGTCTGCGACAG	
Q5SDM17_F	ATTGCTTGCAgAAATTCTCAAAGTTAG	A148G
Q5SDM17_R	GCGGCGTTATAAGCATTTAATG	
Q5SDM18_F	TTTATTTAATtGCATCAATGCATTAAATGCTTATAACGCC	G106T
Q5SDM18_R	GCACCAACGCCTGACTGC	
Q5SDM19_F	CAAGATGGGGtTGGGGCAGTC	A73T
Q5SDM19_R	TCTGCGACAGATTCCTGG	
Q5SDM20_F	TGGCTTATCCGAGGAATCTGTC	C46G
Q5SDM20_R	AGTTCATTTTTCTTTTTTTCATAAATTG	
Q5SDM21_F	GCAGACAAGAgGGGGATGGGG	T68G
Q5SDM21_R	GACAGATTCCTGGGATAAGCC	
Q5SDM22_F	GATGGGGCAGaCAGGCGTTGG	T82A
Q5SDM22_R	CCCATCTTGTCTGCGACAG	
Q5SDM23_F	AAAGCAATTTgTGAAAAAAAGAAAAATGAACTTGGCTTATC	A14G
Q5SDM23_R	AAGGCGACGTGCGTCCTC	
Q5SDM24_F	CTTTATTTAAaGGCATCAATGCATTAAATGCTTATAACG	T105A
Q5SDM24_R	CACCAACGCCTGACTGCC	
Q5SDM25_F	TGCTTATAACaCCGCATTGCTTG	G133A
Q5SDM25_R	TTTAATGCATTGATGCCATTAAATAAAG	
Q5SDM26_F	CGTTGAAGAAcTTAGCCCTTC	T175C
Q5SDM26_R	CTAACTTTGAGAATTTTTGCAAG	
Q5SDM27_F	AGTTAGCGTTaAAGAATTTAGC	G169A
Q5SDM27_R	TTGAGAATTTTTGCAAGC	
Q5SDM28_F	TATAACGCCGaATTGCTTGCA	C137A
Q5SDM28_R	AGCATTTAATGCATTGATGC	
Q5SDM29_F	GCAGACAAGAcGGGGATGGGG	T68C
Q5SDM29_R	GACAGATTCCTGGGATAAGCC	

Lower case letter sin the primer sequences indicate the targeted mutation to incorporate.

Supplementary Table 2. Reference set

ID	category	WT	Substitution	Position
G24A	missense	G	A	24
A25G	missense	A	G	25
A27T	missense	A	T	27
T41G	nonsense	T	G	41
C46G	missense	C	G	46
G49A	missense	G	A	49
A60C	synonymous	A	C	60
T68G	missense	T	G	68
A73T	missense	A	T	73
G78A	synonymous	G	A	78
T68A	missense	T	A	78
T82A	missense	T	A	82
G85A	missense	G	A	85
C95G	missense	C	G	95
G106T	missense	G	T	106
A148G	missense	A	G	148
C156G	synonymous	C	G	156
A157C	missense	A	C	157
G70A,G71C	missense	G,G	A,C	70,71
G124T,C125G	missense	G,C	T,G	124,125
G124A,C125G	missense	G,C	A,G	124,125
G124A,C125A	missense	G,C	A,A	124,125

Supplementary Table 3. Validation set

ID	category	WT	Substitution	Position
A14G	missense	A	G	14
T68C	missense	T	C	68
T105A	missense	T	A	105
G133A	missense	G	A	133
C137A	missense	C	A	137
G169A	missense	G	A	169
T175C	missense	T	C	175
A25G,G133A	missense	A,G	G,A	25,133
G133A,T175C	missense	G,T	A,C	133,175

Supplementary Table 4. Coefficients for linear models to predict GFP signals from enrichment scores

Expression	Intercept α	Sv,o1 β	Sv,o2_trans γ	adj- R2	p-value
Low	7.23***	-0.51***	0.52***	0.96	5.1e-15
High	4.56***	-2.23***	-1.64	0.84	3.6e-9

Significance code P<1e-4 ***

Supplementary Table 5. Covariance of Enrichment scores Sv,o1 and Sv,o2,trans

Expression level	Rep1	Rep2	Rep3
Low	-1.48	-1.95	-1.85
High	-0.16	-0.22	-0.12

Supplementary Table 6. Coefficients to map replicates 1 and 3 to replicate2

	α1	β1	α3	β3
Low	1.5±0.02	0.9±0.02	1.3±0.02	0.9±0.002
High	1.7±0.03	0.8±0.03	0.7±0.04	1.1±0.05

Supplementary Table 7. Configuration states and the energy terms from Ackers' model

CS*i*	Occupied OR	CI dimer (N*i*)	Downstream gene	Total energy (ΔG_{CS})
1	–	0	ON	0
2	OR3	1	ON	ΔG_3
3	OR2	1	OFF	ΔG_2
4	OR1	1	OFF	ΔG_1
5	OR2, OR3	2	OFF	$\Delta G_2 + \Delta G_3 + \Delta G_{co}$
6	OR1, OR2	2	OFF	$\Delta G_1 + \Delta G_2 + \Delta G_{co}$
7	OR1, OR3	2	OFF	$\Delta G_1 + \Delta G_3$
8	OR1, OR2, OR3	3	OFF	$\Delta G_1 + \Delta G_2 + \Delta G_3 + \Delta G_{co}$

Supplementary Table 8. Parameters for CI model, from the literature

K_a	5×10^7	Ackers, 1982
[OR]	10^{-9} mole	Ackers, 1982
ΔG_1	-11.7 kcal	Ackers, 1982
ΔG_2	-10.1 kcal	Ackers, 1982
ΔG_3	-10.1 kcal	Ackers, 1982
ΔG_{co}	-2 kcal	Ackers, 1982
CI fraction folded	0.993	Huang, 1995

Supplementary Table 9. Parameters estimated for modeling regulatory interaction

$[CI_{E,low}]$	$5.5\times10^{-8}M$	Calculated based on $GFP_{wt,low}$
$[CI_{E,high}]$	$8.4\times10^{-7}M$	Calculated based on $GFP_{wt,high}$
