# Supplementary material for: Changes in gene expression predictably shift and switch genetic interactions
Source: Nat Commun. 2019 Aug 29;10:3886. doi: 10.1038/s41467-019-11735-3 (PMC6715729; doi:10.1038/s41467-019-11735-3)
Supplement: Supplementary file 3 — Reporting Summary [file 41467_2019_11735_MOESM3_ESM.pdf]

nature research

Corresponding author(s): Ben Lehner

Last updated by author(s): Jul 26, 2019

Reporting Summary

Nature Research wishes to improve the reproducibility of the work that we publish. This form provides structure for consistency and transparency in reporting. For further information on Nature Research policies, see Authors & Referees and the Editorial Policy Checklist.

Statistics

For all statistical analyses, confirm that the following items are present in the figure legend, table legend, main text, or Methods section.

n/a	Confirmed	
☐	☒	The exact sample size (*n*) for each experimental group/condition, given as a discrete number and unit of measurement
☐	☒	A statement on whether measurements were taken from distinct samples or whether the same sample was measured repeatedly
☐	☒	The statistical test(s) used AND whether they are one- or two-sided<br>*Only common tests should be described solely by name; describe more complex techniques in the Methods section.*
☐	☒	A description of all covariates tested
☐	☒	A description of any assumptions or corrections, such as tests of normality and adjustment for multiple comparisons
☐	☒	A full description of the statistical parameters including central tendency (e.g. means) or other basic estimates (e.g. regression coefficient) AND variation (e.g. standard deviation) or associated estimates of uncertainty (e.g. confidence intervals)
☐	☒	For null hypothesis testing, the test statistic (e.g. *F*, *t*, *r*) with confidence intervals, effect sizes, degrees of freedom and *P* value noted<br>*Give P values as exact values whenever suitable.*
☒	☐	For Bayesian analysis, information on the choice of priors and Markov chain Monte Carlo settings
☒	☐	For hierarchical and complex designs, identification of the appropriate level for tests and full reporting of outcomes
☒	☐	Estimates of effect sizes (e.g. Cohen's *d*, Pearson's *r*), indicating how they were calculated

Our web collection on statistics for biologists contains articles on many of the points above.

Software and code

Policy information about availability of computer code

Data collection	FACSDiva Version 6.1.2 software was used to collect cells based on their GFP signal
Data analysis	Data analysis were performed in R (v 3.3.3). For the sequencing data processing, SABRE software (v 1.000), PEAR software (v 0.9.6),FASTX Toolkit (v 0.0.13) and seqtk tool (https://github.com/lh3/seqtk) were used. For protein structure visualization, PyMOL (v 1.7.6.0) was used. For protein folding stability prediction, FoldX4 software (http://foldxsuite.crg.eu/) was used.

For manuscripts utilizing custom algorithms or software that are central to the research but not yet described in published literature, software must be made available to editors/reviewers. We strongly encourage code deposition in a community repository (e.g. GitHub). See the Nature Research guidelines for submitting code & software for further information.

Data

Policy information about availability of data

All manuscripts must include a data availability statement. This statement should provide the following information, where applicable:

- Accession codes, unique identifiers, or web links for publicly available datasets
- A list of figures that have associated raw data
- A description of any restrictions on data availability

A full data availability statement is included in the manuscript

Field-specific reporting

Please select the one below that is the best fit for your research. If you are not sure, read the appropriate sections before making your selection.

☒ Life sciences ☐ Behavioural & social sciences ☐ Ecological, evolutionary & environmental sciences

For a reference copy of the document with all sections, see nature.com/documents/nr-reporting-summary-flat.pdf

Life sciences study design

All studies must disclose on these points even when the disclosure is negative.

Sample size	Sample size was determined by the number of all possible single and double nucleotide substitutions in the 177 nucleotide long helix-turn-helix domain of CI (531 all possible single nucleotide mutations and 140,184 all possible double nucleotide muations).
Data exclusions	Sequencing data was filtered with the following criteria: 1) Pair-end sequences that were not assembled due to no-matching at the overlap region or unexpected length were discarded. 2) Variants with more than two nucleotide changes were discarded. 3) Variants with less than 100 read counts in the inputs were discarded with the rationale that each cell carried more than a hundred gene copies. 4) Variants with too big variation between biological replicates (standard error of the mean in enrichment scores >1) were excluded from the downstream analysis. 5) Variants observed only in one of the two expression-level experiments were excluded. After filtering, we ended up with all single nucleotide mutations (531) and 1,213 double nucleotide mutations.
Replication	23 nucleotide mutations were validated by comparing results from individual confirmation experiments and from the bulk-sequencing results. All attempts of replication was successful.
Randomization	Samples were grouped by replicates and no other grouping or randomization of samples were performed.
Blinding	Not relevant for this study since the only grouping of the data during the analysis was performed on biological replicates.

Reporting for specific materials, systems and methods

We require information from authors about some types of materials, experimental systems and methods used in many studies. Here, indicate whether each material, system or method listed is relevant to your study. If you are not sure if a list item applies to your research, read the appropriate section before selecting a response.

Materials & experimental systems

n/a	Involved in the study	
☒	☐	Antibodies
☒	☐	Eukaryotic cell lines
☒	☐	Palaeontology
☒	☐	Animals and other organisms
☒	☐	Human research participants
☒	☐	Clinical data

Methods

n/a	Involved in the study	
☒	☐	ChIP-seq
☐	☒	Flow cytometry
☒	☐	MRI-based neuroimaging

Flow Cytometry

Plots

Confirm that:

☒ The axis labels state the marker and fluorochrome used (e.g. CD4-FITC).

☒ The axis scales are clearly visible. Include numbers along axes only for bottom left plot of group (a 'group' is an analysis of identical markers).

☒ All plots are contour plots with outliers or pseudocolor plots.

☒ A numerical value for number of cells or percentage (with statistics) is provided.

Methodology

Sample preparation	Sample preparation steps are listed in the Methods
Instrument	Sorting was performed on FACSAria II SORP and cell fluorescence analysis was performed on LSR Fortesta
Software	FACSDiva Version 6.1.2 software for collection and R software (v 3.3.3) with FlowCore package was used for analysis.

Cell population abundance	A total of about 90 million cells were passed through, with functional fraction taking up about 20 million cells (20 -30% of the total population) sorted into one gate (Output1) and about 5 million cells (3-4% of the total population) sorted into another gate (Output2). Purity from Output1 was about 99% and purity from Output2 was about 59%. Purity was assessed by passing cells through the instrument again after sorting.
Gating strategy	Relevant gating strategies were shown in the Methods.

☒ Tick this box to confirm that a figure exemplifying the gating strategy is provided in the Supplementary Information.
